# Direct and high throughput (HT) interactions on the ribosomal surface by iRIA

**DOI:** 10.1038/srep15401

**Published:** 2015-10-21

**Authors:** Elisa Pesce, Claudia Minici, Jochen Baβler, Ed Hurt, Massimo Degano, Piera Calamita, Stefano Biffo

**Affiliations:** 1 Istituto Nazionale Genetica Molecolare “Romeo ed Enrica Invernizzi”, Milan, Italy; 2 Dept. of Immunology, Transplantation and Infectious Diseases, San Raffaele Research Institute, Milano, Italy; 3 Biochemistry Center of Heidelberg University, Heidelberg, Germany; 4 Dept. of Biosciences, University of Milano, Milan, Italy

## Abstract

Ribosomes function as platforms for binding of other molecules, but technologies for studying this process are lacking. Therefore we developed *i*RIA (*in vitro* Ribosomes Interaction Assay). In approach I, *Artemia salina* ribosomes spotted on solid phase are used for binding picomoles of analytes; in approach II, cellular extracts allow the measurement of ribosome activity in different conditions. We apply the method to analyze several features of eIF6 binding to 60S subunits. By approach I, we show that the off-rate of eIF6 from preribosomes is slower than from mature ribosomes and that its binding to mature 60S occurs in the nM affinity range. By approach II we show that eIF6 binding sites on 60S are increased with mild eIF6 depletion and decreased in cells that are devoid of SBDS, a ribosomal factor necessary for 60S maturation and involved in Swachman Diamond syndrome. We show binding conditions to immobilized ribosomes adaptable to HT and quantify free ribosomes in cell extracts. In conclusion, we suggest that *i*RIA will greatly facilitate the study of interactions on the ribosomal surface.

The classical method for studying ribosomal interaction is the polysomal profile, based on the separation of cellular extracts on sucrose gradients^1^ followed by the collection of fractions and the analysis by precipitation and Western blotting^2^. This superb method, still in use, is based on lengthy technology (lasting days), it can process a limited number of samples (normally, up to 6 gradients each time), and requires hundreds of micrograms of extracts. In addition, the polysomal profile does not allow to measure direct binding of factors to ribosomes. In view of the fact that highly abundant ribosomal proteins may provide a platform for binding several factors, this gap must be overcome. For instance, several ribosomal proteins act as tumor suppressors binding p53, but we do not know if they act on the ribosomal surface^3^. Perhaps the most intriguing case is the one of RACK1. RACK1 was originally identified as a PKC receptor^4^, and later as a 40S ribosomal protein^2^. RACK1 has been suggested to be involved in more than 200 interactions^5^, without ever demonstrating if RACK1 binds its interactors on the ribosome, or independently of. More recently, an high throughput proteomic study has identified a number of proteins that are associated with actively translating ribosomes (Integrin β1, IGF2BP3, MARCKS), using a SILAC-Based approach^6^. Also in this case, it is not known which of these interactions occur at high affinity on the ribosomal platform.

The capability to measure the biochemical properties of the ribosomal apparatus has clinical relevance. Ribosomopathies are inherited diseases in which mutations of genes encoding for proteins belonging to the ribosomal subunits lead to complex syndromes^7^. For instance, mutations of the *sbds* gene leads to Shwachman Diamond syndrome, characterized by pancreatic insufficiency, recurrent infections and increased risk for Acute Myeloid Leukemya^8^. Although ribosomopathies are due to the loss of ribosomal proteins, it has been often problematic to quantify how many ribosomes are produced in mutant cells.

For these reasons we aimed at developing a new method which may be useful in measuring quantitative binding of proteins to ribosomes, in a format allowing rapid binding, high throughput and isolation of modulators. We tested as proof-of-concept ribosomal association in the presence and absence of eIF6, an antiassociation factor binding the 60S^9^, and measured eIF6 binding sites on ribosomes and on extracts from mutant model cells for ribosomopathies. We provide evidence that the method that we have developed, named here *iRIA* is suitable for studying interactions on the ribosomal surfaces and their modulation.

## Results and Discussion

**Figure 1:**
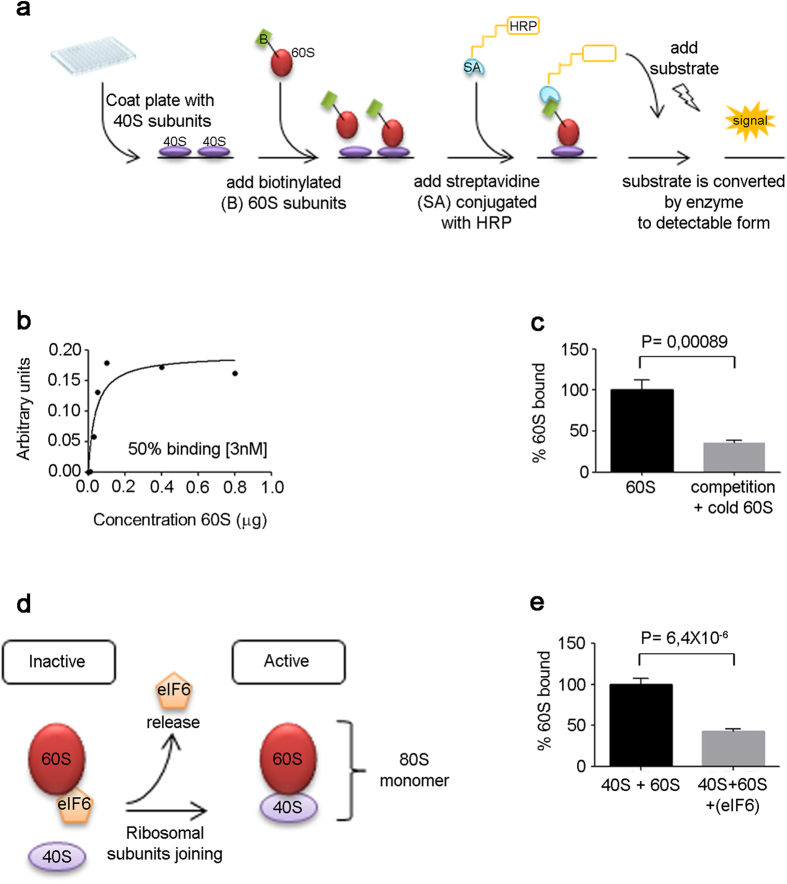
Ribosome-ribosome interactions on a microwell. Feasibility of direct ribosome labeling. (**a**) Experimental design with immobilized 40S subunits and labeled 60S subunits. **(b)** Saturation curve of 60S binding to 40S performed at room temperature for 60 minutes **(c)** 50% displacement by 5-fold unlabeled 60S at room temperature for 10 minutes. **(d)** Scheme showing eIF6 binding to 60S that impairs 80S (40S + 60S) formation. **(e)** eIF6 inhibits binding of 60S to 40S. Here eIF6 was added to labeled 60S subunits for 10 minutes, before the incubation to adsorbed 40S. All experiments were performed at least five times. Data are expressed as means + SD. P-values (P) are indicated. T-test, two-tailed.

The goal of *iRIA* is to measure interactions at the ribosomal surface. We therefore used *iRIA* to answer two unresolved issues as proof-of-concept: to characterize eIF6 binding to 60S subunits and the number of ribosomes present in a cell after SBDS loss. Briefly, the *i*RIA approach for studying the interactions between ribosomes and regulatory factors consists in the development of a platform, in the format of 96-well, containing the purified ribosomal subunits. *i*RIA can arrange labeling of multiple components, including ribosomes, as well as interactions both in liquid and semi-solid phase. Both will be described, with control examples. *Artemia salina*’s cysts contain naturally large amounts of ribosomes in dehydrated form. We reasoned that this organism might be a cheap and high-yield source of ribosomes, resistant to semi-dry conditions. *Artemia salina* ribosomes were purified (Supplementary Method; Supplementary Fig. 1) and their quality was tested by an association assay. Functional 40S and 60S subunits should form 80S dimers^2^ by raising Mg^2+^. *Artemia salina* ribosomes form 80S at 100% efficiency, as shown by sucrose gradient analysis (Supplementary Fig. 1). To increase the flexibility of *i*RIA, in a first variant, we labeled functional ribosomal subunits (Supplementary Fig. 1; Supplementary Method). Although *Artemia salina* ribosomes are inexpensive and suitable for HT technologies, comparable results were obtained by different sources, such as yeasts or mammalian cells. In a first battery of tests, we coated a 96-well plate with 40S subunits. 40S immobilized in the well was incubated with labeled 60S subunits (Fig. 1a). We observed a saturable binding in the nanomolar range (Fig. 1b). The binding was displaced by adding an excess of unlabeled 60S subunits showing its specificity (Fig. 1c; Supplementary Fig. 1). Labelling of 40S and binding to immobilized 60S was also effective (Supplementary Fig. 1), but was not further explored because we focused on eIF6–60S interaction.

eIF6 is a rate-limiting translation and ribosome binding factor that is necessary for cancer progression^10^,^11^. eIF6 prevents 80S association^7^ *in vitro* and *in vivo,* by binding to 60S as in^2^ (scheme: Fig. 1d). Recombinant full length eIF6 was produced in the presence of a chaperone mixture^12^ and purified as described (Supplementary methods). We first validated the binding of eIF6 to *Artemia salina* 60S ribosomes in a traditional equilibrium experiment (Supplementary Fig. 2). Data show that in the presence of 60S ribosomes eIF6 shifts to the heavy part of the gradient, as expected. Rapid freezing of eIF6 leads to loss of eIF6 activity that is unable to bind 60S ribosomes, although still in the soluble phase (Supplementary Fig. 2). Next, we tested whether eIF6 anti-association activity was effective in our *i*RIA. First, eIF6 blocked the binding of labeled 60S subunits to immobilized 40S (Fig. 1e; Supplementary Fig. 2). Excess of two unrelated proteins (instead of eIF6), pyruvate dehydrogenase (PDH) and SBDS, the latter necessary for ribosomal maturation^13^ did not prevent binding of labeled 60S to immobilized 40S (Supplementary Fig. 2). This first set of experiments demonstrates the feasibility of labeling ribosomes and handling them on a microwell. The fact that SBDS was, by itself, unable to inhibit eIF6 binding to 60S, even if involved in its release^14^, confirms the model that it might be necessary but not sufficient^9^.

**Figure 2:**
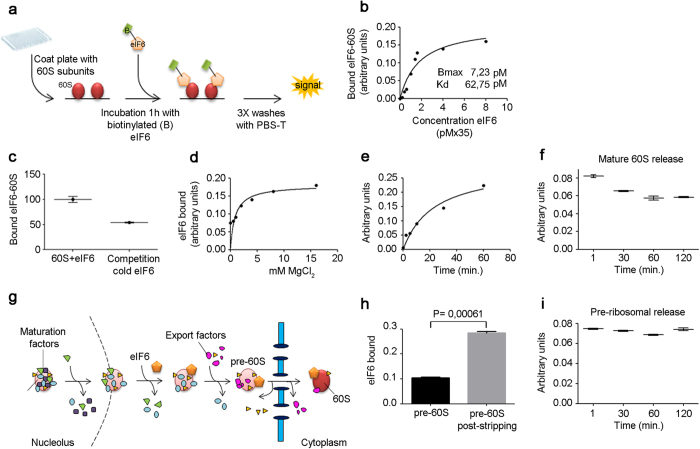
Protein-ribosome interaction on a microwell. eIF6 off-rate is different in pre-ribosomes and mature ribosomes.(**a**) Experimental design with immobilized 60S subunits and labeled eIF6. **(b)** Saturation curve of eIF6 binding to 60S. Binding performed at room temperature. **(c)** 50% displacement by ten-fold unlabeled eIF6 coincubated with labeled eIF6. **(d)** Dependence of eIF6 binding to 60S by Mg^2+^. **(e)** On-rate. **(f)** Off-rate of eIF6 from mature ribosomes, shows eIF6 release in the first 30 min. All the experiments b-f were performed at least five times. Mean + SD. **(g)** Scheme of ribosomal maturation with eIF6/Tif6 bound both to pre-ribosomes in the nucleus and mature ribosomes in the cytoplasm. eIF6 is added to preribosomes in the nucleolar/nucleus compartment and exported to the cytoplasm. In the cytoplasm 60S subunits are inactive until eIF6 is released. **(h**) Stripping of endogenous eIF6 from purified adsorbed pre-ribosomes leads to increased exogenous eIF6 binding to pre-ribosomes. eIF6 binding to nucleolar ribosomes before (black) and after (grey) stripping of endogenous eIF6 with 300 mM KCl. **(i)** eIF6 off-rate from pre-ribosomes, loaded with exogeneous eIF6 after stripping of the endogenous one. All the experiments were performed at least five times. Representative curves are shown. Mean + SD. T-Test, paired, two-tailed.

Next, we proceeded to develop quantitative binding of proteins to immobilized ribosomes (scheme: Fig. 2a). We used eIF6 binding to 60S to both validate the method and to answer specific questions. We immobilized 60S subunits and bound labeled eIF6 (Supplementary methods). eIF6 binding was saturable (Fig. 2b). Fifty percent inhibition of labeled eIF6 was obtained with competition with “cold” eIF6 (Fig. 2c; Supplementary Fig. 3). The method was characterized by very low SD. In addition, the signal was strictly depending on 60S ribosomes adsorbed in the well, and all components present (Supplementary Fig. 3). No binding of eIF6 occurred to BSA and BSA + KCl, and only minimal background was observed from binding of avidin to immobilized 60S subunits (Supplementary Fig. 3). To establish whether *iRIA* is highly reliable, we calculated, in according to NCGC assay guidance^15^, the parameters to satisfy for an High Throughput Screening by focusing on the reaction eIF6 + 60S → [eIF6:60S]. The variability tests were conducted on three types of signals: Max, Min, Mid, run over 3 days to assess uniformity and separation of signals, and using 2% DMSO in the mixture. We obtained a **Z**-factor value of 0.82, indicating robustness of this assay. We then further proceeded to the characterization of eIF6 binding to 60S. Mg^2+^ increases 60S rigidity. eIF6 bound mature 60S at concentrations of Mg^2+^ as low as 0.5 mM, corresponding to free intracellular Mg^2+^ (Fig. 2d). By increasing Mg^2+^ eIF6 binding becomes tighter, suggesting that it can be modulated by allosteric changes in 60S. At low Mg^2+^ 25% percent of maximal eIF6 binding occurs within two minutes in the pM range (Fig. 2e). After achieving maximal binding, we calculated the off-rate of eIF6 from mature 60S ribosomes; the binding was fairly stable, with about 20% of eIF6 released at 30 min (Fig. 2f). Taken together our observations suggest that eIF6 can bind mature ribosomes at high efficiency and rapidity, in agreement with *in vivo* observations^2^. Data suggest however that some mature ribosomes have a conformation that require additional event to efficiently release eIF6, whereas some can arrange rapid on-off exchanges.

eIF6 is necessary for 60S biogenesis and binds not only mature 60S subunits, but also pre-60S ribosomes in the nucleus (scheme: Fig. 2g and ref. 9). We prepared pre-ribosomes from *S. cerevisiae* extracts (Supplementary Fig. 4), which normally have tightly bound the eIF6 homolog Tif6^16^. A late cytoplasmic pre-ribosomes, purified via the biogenesis factor Yvh1, was immobilized in the 96-well plate. We measured eIF6 binding both on the native preribosome and in the preribosome in which endogenous Tif6 was stripped through a transient treatment with 300 mM KCl. Incubation with labeled eIF6, after stripping with KCl and reequilibration in physiological conditions, resulted in the expected increase of signal (Fig. 2h, control of BSA, Supplementary Fig. 3). The signal is saturable (Supplementary Fig. 4). Next, we measured the off-rate of eIF6 from pre-ribosomes (as in Fig. 2f). No release was seen even at 120 min after wash (Fig. 2i). Taken together data demonstrate that preribosomes stably bind eIF6 in the absence of a release factor.

**Figure 3:**
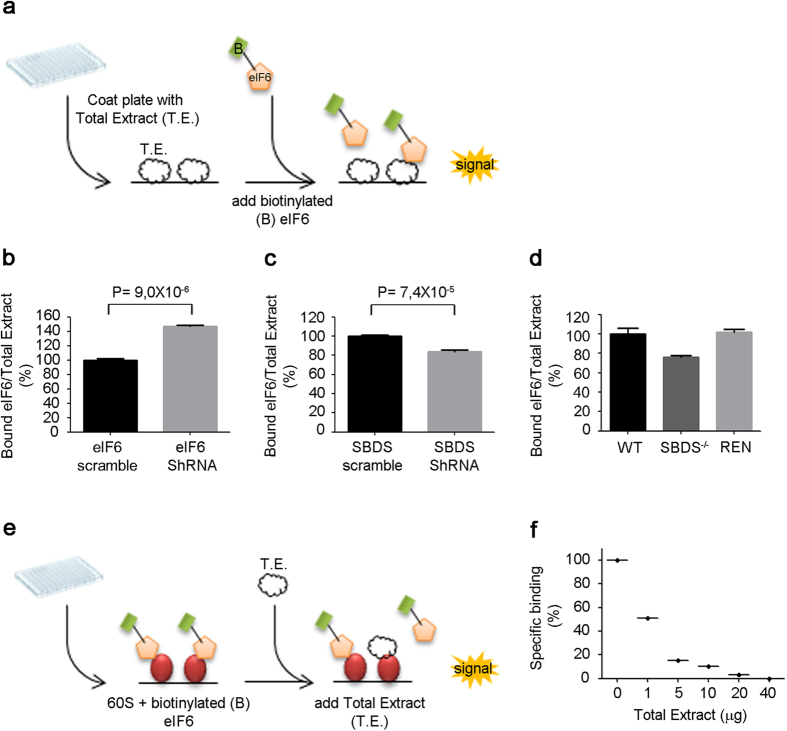
Measuring ribosomal binding sites on total extracts. SBDS depletion reduces eIF6 binding sites. **(a)** Experimental design with immobilized extracts and labeled eIF6. **(b)** *In vivo* eIF6 sh-RNA increases *in vitro* available 60S sites. Equal amounts of extracts from either control cells (scramble, black) or eIF6-depleted cells (eIF6 ShRNA, grey) were incubated with labeled eIF6. (**c**) *In vivo* SBDS depletion decreases *in vitro* available 60S sites. Experimental design as in b). **(d)** SBDS-deficient fibroblasts have less binding sites for eIF6, respect to wt and REN cell line^24^. In this case we used a cell line with genetically inactivated SBDS (SBDS −/−) and we compared it to a matched wt or to a mesothelioma cell line (**e)** Design of activity evaluation for eIF6 release. **(f)** eIF6 is released by cellular extracts. After having performed total eIF6 binding on immobilized ribosomes, given amounts of extracts were added, and residual eIF6 binding was measured. All the experiments were performed at least five times. Mean + SD. T-Test, paired, two-tailed.

Having established that iRIA may work with different purified ribosomal sources, we attempted at adapting it to simple protein extracts. We therefore spotted cellular extracts and measured eIF6 binding (Fig. 3a). In this case, the major problem would be to show that eIF6 binding can accurately measure free ribosomal 60S number. We exploited, as proof-of-concept the idea that eIF6 depletion decreases the amount of eIF6 on 60S complex, and leads to an increase in free 60S number*. In vivo,* the absence of eIF6 binding to 60S leads to the formation of unstable, inactive 80S (vacant, without mRNA) couples^10^ which can be dissociated by increasing salt conditions. Indeed, we demonstrate that cellular extracts depleted *in vivo* by eIF6 have an increase of eIF6 binding *in vitro* (Fig. 3b) and an increase of 80S *in vivo* (Supplementary Fig. 5). In conclusion, eIF6 depletion leads to an increase of 80S due to the loss of eIF6 antiassociation activity that can be precisely measured by our assay through the binding of eIF6 to 60S ribosomes solubilized from cellular extracts. Last, we applied this method to another condition in which ribosome number might change. It is a matter of debate if in ribosomopathies the actual number of ribosomes drop, due to the paucity of methods to analyse it. Shwachman-Diamond Syndrome is caused by loss of SBDS^8^, and may involve failed eIF6 release^17^. We used *iRIA* to measure whether SBDS depletion (Supplementary Fig. 5) leads to a reduction of 60S binding sites. We show that in human cells, SBDS-depleted extracts, by SBDS sh-RNA, show a reduction in eIF6 binding (Fig. 3c) compared to controls. Strikingly, identical results were obtained assaying 60S binding sites on a totally independent mouse fibroblast line derived from a Swachman-Diamond model^18^, compared to wild type fibroblasts and a control cell line (Fig. 3d). In another setup, we measured eIF6 binding sites on cytoplasmic and nuclear ribosomes. We show that 60S cytoplasmic binding sites are three-fold higher than nuclear (Supplementary Fig. 6), even if more eIF6 is present in the cytoplasm (Supplementary Fig. 6). Last, we evaluated whether we could use the system to study dynamic properties of ribosome binding such as release. In this setup (Fig. 3e) we can spot a given ribosomal preparation, incubate with an analyte and modulate it by biochemical preparations. We spotted 60S subunits, incubated with eIF6 in the presence of cellular extracts with normal eIF6 levels. We show, by titration, that cellular extracts have an eIF6 release activity (Fig. 3f). The release activity does not depend on competition with endogenous eIF6 levels because it is observed also with extracts depleted from eIF6 (Supplementary Fig. 6). The nature of this activity remains to be determined.

Our goal was to develop the first method for quantitative and efficient binding of proteins to ribosomes. Indeed, we provide a versatile method, *i*RIA, for studying ribosomal-interactions. This method allows the quantitative binding of proteins to pure ribosomes and measurement of kinetic parameters, as well as to use the method for screening modulating compounds with high Z-factor. *i*RIA can be adapted by ribosomal labeling techniques. We provide strategies to adapt *i*RIA for measuring the relative amount of ribosomes in different cellular extracts. By *i*RIA, we provide proof that i) eIF6 binds both mature and pre-ribosomes at high affinity, but that eIF6 off-rate form pre-ribosomes is virtually null in the absence of modulating factors, ii) extracts have a clear eIF6-release activity and iii) free 60S ribosomes are reduced in cells devoid of SBDS; iv) eIF6 depletion leads to the concomitant increase of 80S *in vivo* due to the loss of eIF6 antiassociation activity that can be quantified through iRIA of around 40%, very close to the 50% reduction of eIF6 levels by shRNA. These last observations provide a mechanistic insight on the fact that 50% eIF6 depletion as obtained in eIF6 het. mice results in decreased growth factor stimulated translation^11^. The appearance of several non-translated RNAs which can modulate translation by binding target molecules, raise the question on whether they may directly bind ribosomes or not^19^. IRES-containing RNA viruses bind ribosomes without the assistance of Initiation Factors^20^. A technology that may lead to the identification of RNAs directly binding ribosomes, like *i*RIA, before sequencing them may help to solve also this issue.

## Methods

### Ribosomes purification

A procedure modified from Zasloff and Ochoa^21^ was used for preparing *Artemia salina* ribosomes. A detailed step-by-step protocol can be found in the supplemental file.

### Labelling of 60S subunit

The amount of biotin that was used for each reaction was calculated by using an appropriate molar ratio of biotin to ribosomes. In our experiments it was for 60S, 400 μg/ml of RNA and 0,00026 mM of biotin and for 40S, 400 μg/ml of RNA and 0,0005 mM of biotin. In general, we used a standard protocol from the biotin manufacturer (EZ-Link® NHS-Biotin, Pierce). Purified ribosomes were dissolved in PBS (137 mM NaCl, 2,7 mM KCl, 10 mM Na_2_HPO4, 1,8 mM KH_2_PO_4_ adjusted pH to 7,4 ) and incubated with the appropriate volume of biotin reagent solution for two hours at 4 °C in a rotating wheel. Labeled ribosomes were dialyzed against PBS O/N at 4 °C to remove non-reacted biotin.

### eIF6 expression, purification and labeling

To test the binding activity of ribosomes, the human full length N-terminal his-tagged eIF6 was expressed in a bacterial system. We achieved suitable levels of soluble eIF6 protein exploiting the BL21(DE3) strain of E. coli previously transformed with different vectors encoding for the bacterial molecular chaperons DnaK, DnaJ, GrpE, ClpB, and GroESL^12^. A detailed step-by-step protocol can be found in the supplemental file.

### Biochemical purification of pre-60S particles

The S. cerevisiae strain DS1-2b (MAT alpha, leu2, his3, trp1, ura3) was used to tag the YVH1 gene with proteinA-TEV-FLAG tag at its C-terminus^22^.Yeast cells were grown in 2l YPD liquid cultures to OD 2,8 at 30 °C and mechanically lysed in a cryo mill (Retsch). Supernatant of the cell lysate was incubated first IgG Sepharoese (GE Health care) to isolate Yvh1 and associated pre-ribosomes. Elution was done by incubation with TEV protease. This eluate was further purified by incubation with anti-FLAG beads (Sigma-Aldrich) and finally eluted with excess of FLAG peptide. For details^23^.

### *i*RIA assay

Protocol Used for 96-well plates. All amounts refer to a single well. 96-well plate was coated with 3 nM purified ribosomes diluted in 50 μl of PBS, 0,01% Tween-20, O/N at 4 °C in humid chamber. Coating solution was removed and aspecific sites were blocked with 10% BSA powder in PBS, 0,01% Tween-20 for 30 minutes at 37 °C. Plate was washed with 100 microliters/well with PBS-Tween. 0,5 μg of recombinant biotynilated eIF6 was resuspended in a reaction mix: 2,5 mM MgCl_2_, 2% DMSO and PBS-0.01% Tween, to reach 50 μl of final volume/well, added to the well and incubated with coated ribosomes for 1 hour at room temperature. To remove unbound protein, each well was washed 3 times with PBS-Tween. HRP-conjugated streptavidine was diluted 1:7000 in PBS-Tween and incubated in the well, 30 minutes at room temperature, volume 50 μl. Excess of streptavidine was eliminated through three washes with PBS-Tween. OPD (*o*-phenylenediamine dihydrochloride) was used in according to the manufacturer’s protocol (Sigma-Aldrich) as a soluble substrate for the detection of streptavidine peroxidase activity. The signal was detected after the incubation, the plate was read at 450 nm on a multiwell plate reader (Microplate Bio-Rad model 680).

### Polysomal profile

Purified subunit 40S and 60S were separated on a 10–30% sucrose gradient under conditions not favorable to the association of 80S (1 mM Mg^2+^), 50 mM Tris-acetate (pH 7.5), 50 mM NH_4_Cl, 1 mM MgCl_2_ and 1 mM DTT or under standard conditions 50 mM Tris-acetate (pH 7.5), 50 mM NH_4_Cl, 12 mM MgCl_2_ and 1 mM DTT. Absorbance at 254 nm was recorded by BioLogic LP software (BioRad). and fractions collected. Fractions were precipitated with 10% trichloroacetic acid (TCA) according to standard protocol, separated on SDS-PAGE and analyzed by Western blot.

### Mammalian cell culture and lentiviral infection

HEK-293T and HeLa cells from ATCC were cultured in DMEM (Euroclone) supplemented with 10% fetal bovine serum (FBS) and penicillin/streptomycin/glutamine solution (GIBCO) at 37 °C under 5% CO_2_. Mycoplasma testing was performed before experiments. 293T cells were transfected at 60–70% confluence with pGIPZ eIF6 shRNA and pGIPZ scramble lentiviral vectors (second generation lentivirus from Open Biosystem) and with pFCY, SBDS shRNA and pFCY scramble lentiviral vector (a generous gift from the lab of DC. Link, Washington University School of Medicine) to produce lentiviral particles. HeLa cells were infected with lentivirus at a confluence of 50%. Experiments were performed after one week from infection. Silencing of protein was measured by Western Blot analysis.

SBDS cell lines were generated in our lab (Calamita P. *et al.*, in preparation) by immortalizing MEFs with DNp53Ras retroviral vectors. The mutated line express in homozygosity the SBDS protein with the R126T point mutation. Primary MEFs belong to the SBDS mouse model of the laboratory of Joahanna Rommens (The Hospital of Sick Children, Toronto).

### Data Analysis

Plate uniformity and signal variability of *i*RIA were assessed by calculating of the Z’-factor, coefficient of variation (CV) and signal window (SW).

For new assays the plate uniformity study should be run over 3 days to assess uniformity and separation of signals, using DMSO at the concentration to be used in screening. Z’-factor statistic was determined by using minimum signal as background and maximum signal as positive control. Mid-signal wells were computed a 50% activity relative to the means of the max. Three independent experiments were performed. The Z’ factor was calculated using the equation Z’ = 1–3 (SD_positive_ + SD_background_)/(AVG_positive_ – AVG_background_).

For all experiments, statistical analysis was performed with GraphPad Prism 5.

### Nuclear and cytoplasmic fractionation

Nuclear and cytoplasmic extract were prepared using the NE-PER^®^ Nuclear and Cytoplasmic Extraction reagents (Thermo Scientific) as per manufacturer’s instruction. Protein concentration was quantified using the BCA protein assay kit (Pierce). Equal amounts of protein were loaded in each lane and separated on a 10%, 12% SDS-PAGE, then transferred onto a nitrocellulose membrane. Membranes were probed with the following primary antibodies: mouse anti-eIF6 monoclonal, rabbit anti-Tubulin (1:1000 Cell Signaling), mouse anti-LaminB (1:1000 Santa Cruz), mouse β-actin.

## Additional Information

**How to cite this article**: Pesce, E. *et al.* Direct and high throughput (HT) interactions on the ribosomal surface by iRIA. *Sci. Rep.* **5**, 15401; doi: 10.1038/srep15401 (2015).

## Supplementary Material

Supplementary Information
