# Author Correction: Direct and high throughput (HT) interactions on the ribosomal surface by iRIA

**DOI:** 10.1038/s41598-023-28876-7

**Published:** 2023-02-02

**Authors:** Elisa Pesce, Claudia Minici, Jochen Baßler, Ed Hurt, Massimo Degano, Piera Calamita, Stefano Biffo

**Affiliations:** 1 Istituto Nazionale Genetica Molecolare “Romeo Ed Enrica Invernizzi”, Milan, Italy; 2 Dept. of Immunology, Transplantation and Infectious Diseases, San Raffaele Research Institute, Milan, Italy; 3 Biochemistry Center of Heidelberg University, Heidelberg, Germany; 4 Dept. of Biosciences, University of Milano, Milan, Italy

Correction to: *Scientific Reports* 10.1038/srep15401, published online 21 October 2015

The original version of this Article contains an error in the spelling of the author Jochen Baßler which is incorrectly given as Jochen Baβler.

