# Supplementary material for: Direct and high throughput (HT) interactions on the ribosomal surface by iRIA
Source: Sci Rep. 2015 Oct 21;5:15401. doi: 10.1038/srep15401 (PMC4613909; doi:10.1038/srep15401)
Supplement: Supplementary Information [file srep15401-s1.pdf]

Title: Direct and high throughput (HT) interactions on the ribosomal surface by iRIA

Authors: Elisa Pesce[1], Claudia Minici[2], Jochen Baβler[3], Ed Hurt[3], Massimo Degano[2], Piera Calamita[1], and Stefano Biffo[1,4]

[1]Istituto Nazionale Genetica Molecolare "Romeo ed Enrica Invernizzi", Milan, Italy
[2]Dept. of Immunology, Transplantation and Infectious Diseases, San Raffaele Research Institute, Milano, Italy
[3]Biochemistry Center of Heidelberg University, Heidelberg, Germany
[4]Dept. of Biosciences, University of Milano, Milan, Italy

SUPPLEMENTARY METHODS

Ribosomes purification

In detail, 50 g of *Artemia* cysts were suspended in 100 ml of ice-cold 20% sodium hypochlorite. The cysts were left in this solution for 5 minutes, shaking every minute once. To remove the bleach, cysts were filtered at 0.4 microns, washed twice with cold sterile water and transferred into a dry becker. Note that in water, viable cysts sink to the bottom of the becker, while those that remain in suspension have either the cortex damaged or are died. Procedure to work, all cysts must be viable. The cysts were then subjected to 6 cycles of washings with 500 ml of cold sterile water, by cycles of mixing and sedimentation for about 15 minutes each time. The particulate on the surface was drained away at the end of each wash. After a final filtration, the cysts were transferred from the filter to a pre-cooled mortar and grinded with 37.5 g of glass beads with the gradual addition of 20 ml of buffer A (70 mM KCl, 1 mM DTT, 9 mM $MgCl_2$, 0.1 mM EDTA, 5% glycerol, 20 mM Tris HCl, pH7.5) until they reached a fine yellowish texture. Then, 60 ml of buffer A were added to obtain a homogeneous suspension, which was filtered through four layers of sterile cheesecloth (I). The solid paste that was retained by the cheesecloth was transferred again in the mortar and re-grinded with the gradual addition of 40 ml buffer A. The slurry was filtered through four layers of sterile cheesecloth (II). The resulting filtrates (step I + II) were centrifuged in Beckman centrifuge with Allegra 2IR 30 ml corex at 10,733 g, 30 minutes at 4°C. The supernatant was filtered with poly-prep chromatography columns (Bio-Rad) avoiding to collect the orange layer of lipids, and centrifuged at 71,256 g for 20-30 minutes at 4°C with a Beckman 50.2 Ti rotor. The supernatant was re-filtered again and centrifuged at 145,421 g for 2 hours and 30 minutes at 4°C with the rotor 50.2Ti. The obtained pellet is formed by ribosomes. The ribosomal pellet was washed gently with buffer A and then re-suspended in 0.5 ml of buffer B (2 mM DTT, 4 mM $MgCl_2$, 700 mM KCl, 20 mM Tris HCl, pH 7.5). The resuspension was loaded into 15-30% linear sucrose gradients in 20 mM Tris-HCl (pH7.5), 2 mM DTT, 11 mM $MgCl_2$, 700 mM KCl and ultracentrifuged on a SW41 Ti for 16 hours at 49,392 g, 4°C. The fractions, corresponding to either the isolated 40S or 60S subunits were pooled and diluted with 20 mM Tris HCl (pH7.5), 2 mM DTT, 2 mM $MgCl_2$ to reach a final concentration of 100 mM KCl. Next, they were concentrated with Centricon YM100 at 2500-3000 g (Megafuge Heraeus 1.0R) to remove sucrose and were quantified by spectrophotometer (Abs 260λ). In our hands, final yield was 661 μg of RNA for 60S and 484 μg of RNA for 40S subunits. Both subunits were brought to 50% glycerol, divided into working aliquots and stored at -80°C. Stability is at least one year.

eIF6 expression, purification and labelling

In detail, the culture was grown under antibiotic selection at 37°C until the optical absorption at 600 nm reached a value of 0.8. Cells were then harvested, resuspended in fresh medium, and protein expression was induced by addition of IPTG to a final concentration of 0.25 mM. Cells were incubated 16 hours at 20°C before being harvested by centrifugation at 8000g. Cell pellet was resuspended in 1/25th of culture volume with a buffer consisting of 50 mM Tris (pH 8), 300 mM NaCl, 10 mM Imidazole, 1 mM β-mercaptoethanol, 1 mg/ml Lysozyme, 2 μg/ml DNase, 20 μg/ml RNase A and complete EDTA-free protease inhibitor cocktail (Roche). Cells were disrupted by sonication and the soluble phase was separated from inclusion bodies and cellular debris by a centrifugation at 25000 g for 45 minutes at 4°C. eIF6 was at first purified by affinity chromatography. Cleared cell lysate was loaded onto the Ni-NTA matrices at a ratio of 50:1 and incubated for one hour at 4°C under gentle agitation. The resin was extensively washed with the resuspension buffer and a wash buffer consisting of 50 mM Tris (pH 8), 1 M NaCl, 10 mM Imidazole, and 1 mM β-mercaptoethanol to remove the untagged proteins. Elution was obtained incubating the resin with the elution buffer (50 mM Tris (pH 8), 300 M NaCl, 300 mM Imidazole,

and 1 mM β-mercaptoethanol). The affinity-purified protein was then dialyzed against a buffer containing 50 mM Hepes (pH 7.6), 500 mM NaCl at 4 °C for 16 hours and further purified by a size exclusion chromatography on a Superose 12 10/300 column (GE Healthcare) operated with the AktaPurifier10 FPLC system. The protein containing fractions were collected, concentrated with a Vivaspin 20 ml concentrator (Sartorius), and analysed on a denaturing 10% SDS-PAGE to evaluate purity level. Recombinant eIF6 was labelled with biotin in according to standard protocol (EZ-Link® NHS-Biotin, Pierce). In our experiments 600 µg/ml of recombinant eIF6 were labeled with 0,26 µM of biotin.

SUPPLEMENTARY FIGURES 1-6

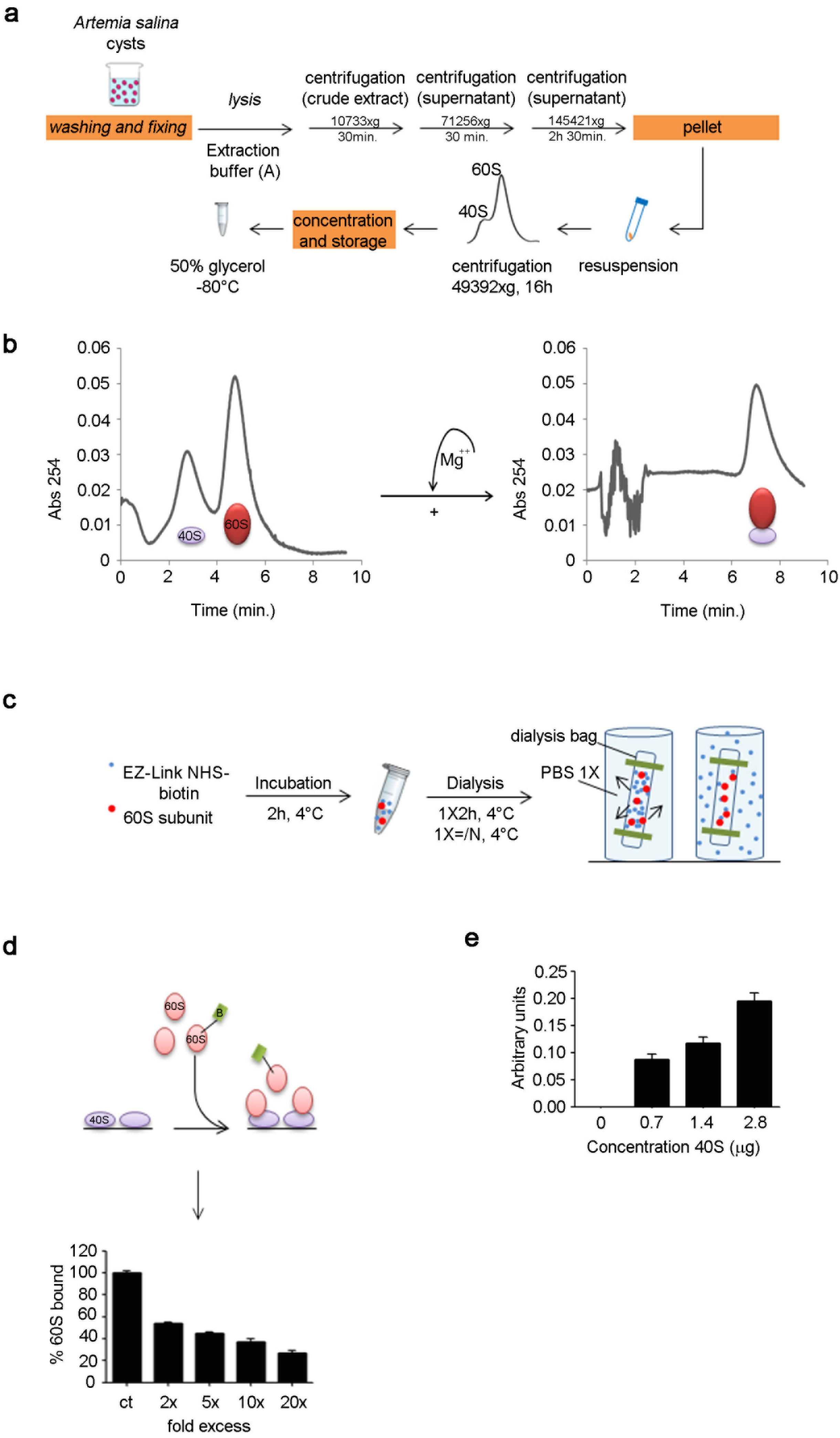
a
Artemia salina cysts
washing and fixing
lysis
Extraction buffer (A)
centrifugation (crude extract)
10733xg 30min.
centrifugation (supernatant)
71256xg 30 min.
centrifugation (supernatant)
145421xg 2h 30min.
pellet
resuspension
60S
40S
centrifugation 49392xg, 16h
concentration and storage
50% glycerol -80°C
b
Abs 254
Time (min.)
40S
60S
Mg++
+
c
EZ-Link NHS-biotin
60S subunit
Incubation
2h, 4°C
Dialysis
1X2h, 4°C
1X=/N, 4°C
dialysis bag
PBS 1X
d
60S
B
40S
% 60S bound
ct
2x
5x
10x
20x
fold excess
e
Arbitrary units
Concentration 40S (μg)

Supplementary Figure 1. Purification and labeling of ribosomal subunits. **(a)** Scheme of purification of the 60S and 40S ribosomal subunits from *Artemia salina* cysts. **(b)** Mg^{2+} induces the association of the 40S and 60S ribosomal subunits to form the 80S. Salt-washed ribosomal subunits remain dissociated in 1 mM Mg^{2+} (left panel), but associate after raising the concentration to 5 mM Mg^{2+} (right panel). **(c)** Scheme of biotinylation of 60S subunits. **(d)** Increasing concentration of unlabeled 60S (1x to 20x fold excess) inhibiting the binding of labeled 60S to immobilized 40S. **(e)** Experiment performed in the linear range of binding shows that biotinylated 40S binds immobilized 60S. All experiments were performed at least five times except the one in Figure e which was done twice. Mean + SD are shown.

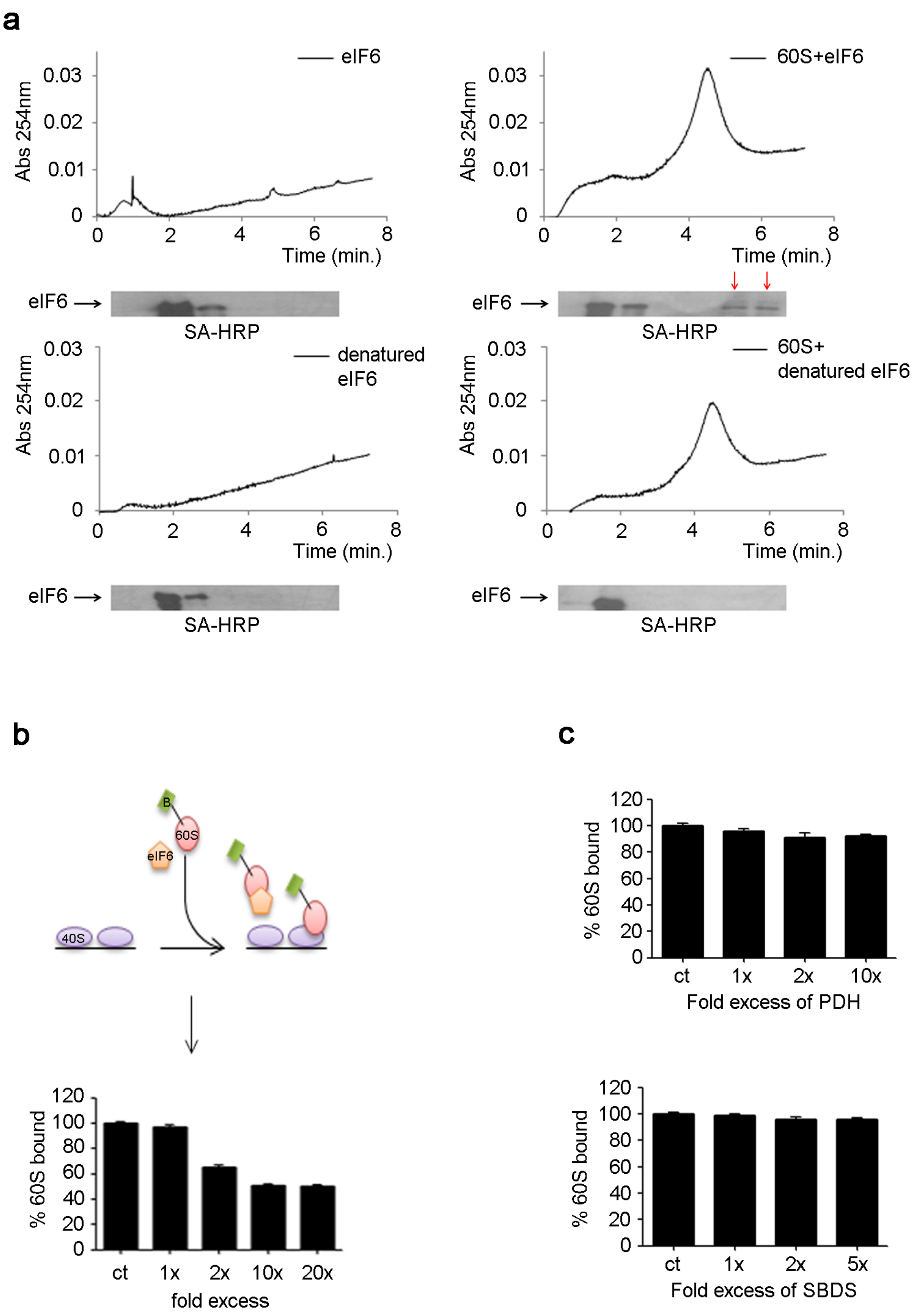

Supplementary Figure 2. Recombinant biotinylated eIF6 binds purified 60S subunit. (a) eIF6 alone (top left) or after incubation with 60S subunit (top right). All samples are loaded on 15-30% sucrose gradient. Absorbance is measured at 254 nm. The collected fractions were concentrated with TCA, run on 12% gel SDS-PAGE and analyzed by W.B. To detect biotinylated eIF6, we used streptavidine conjugated with peroxidase. Bottom left, denatured eIF6 without ribosomes. Bottom right, denatured eIF6 with ribosomes. Note that eIF6 shift occurs only with native eIF6 on ribosomes (top right, red arrows). (b) Increasing concentration of unlabeled eIF6 (1x to 20x fold excess) inhibits the binding of labeled 60S to immobilized 40S. Control (ct) value is without competing eIF6. (c) Labelled 60S was mixed with an excess of Pyruvate Dehydrogenase or SBDS. No inhibition of binding is observed. All experiments were performed at least five times. Mean + SD are shown.

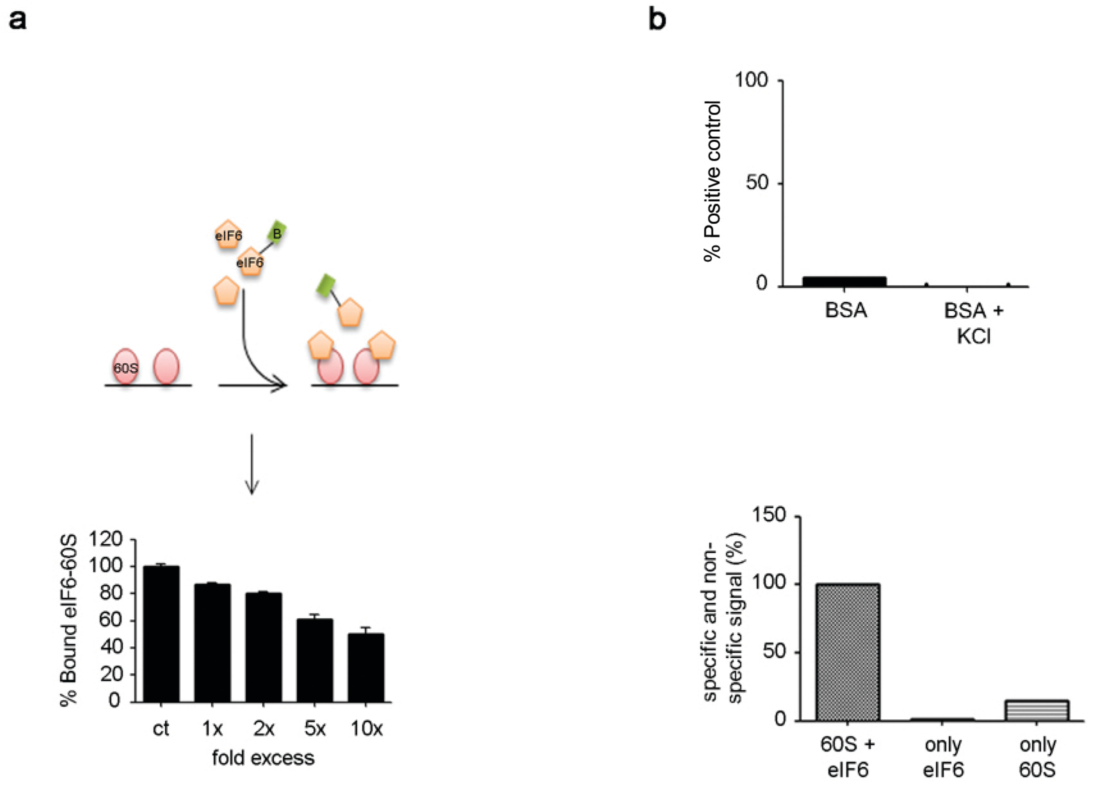

Supplementary Figure 3. Dose-dependent competition analysis. (a) 60S is coated in the well. Labeled eIF6 is added in the presence of the indicated amounts of unlabeled eIF6. All experiments were performed at least five times. Mean + SD are shown. (b) Specific and non-specific binding in iRIA. The chart shows background staining of eIF6 without ribosomes, ribosomes without eIF6, or unrelated protein BSA on ribosomes before or after stripping with KCl. Mean + SD are shown, SD less than 3%.

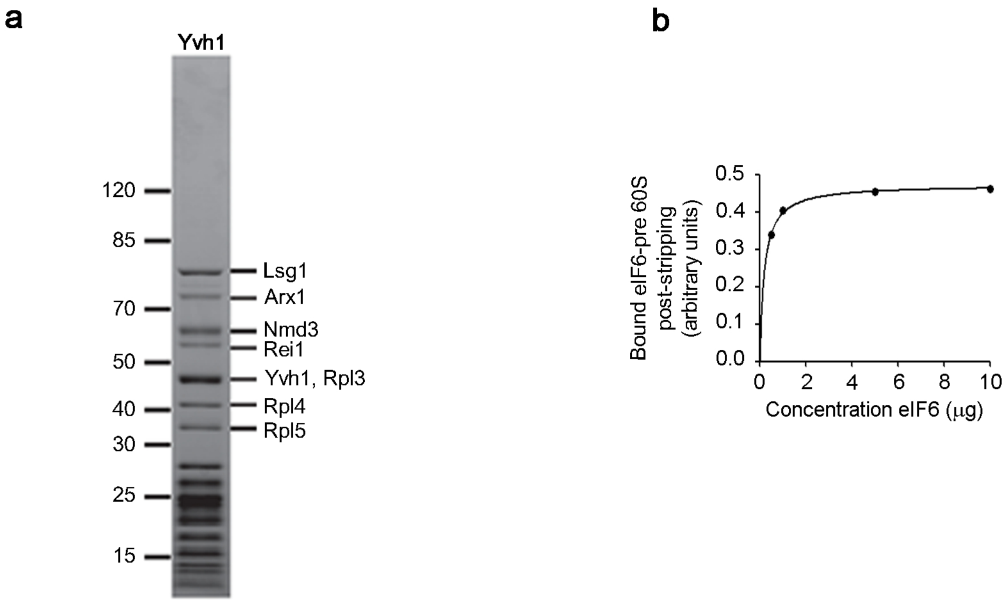

Supplementary Figure 4. Binding of eIF6 to preribosomes. (a) Coomassie staining of purified pre-ribosomes. (b) Saturation curve on preribosomes. All experiments performed at least five times. SD less than 5%.

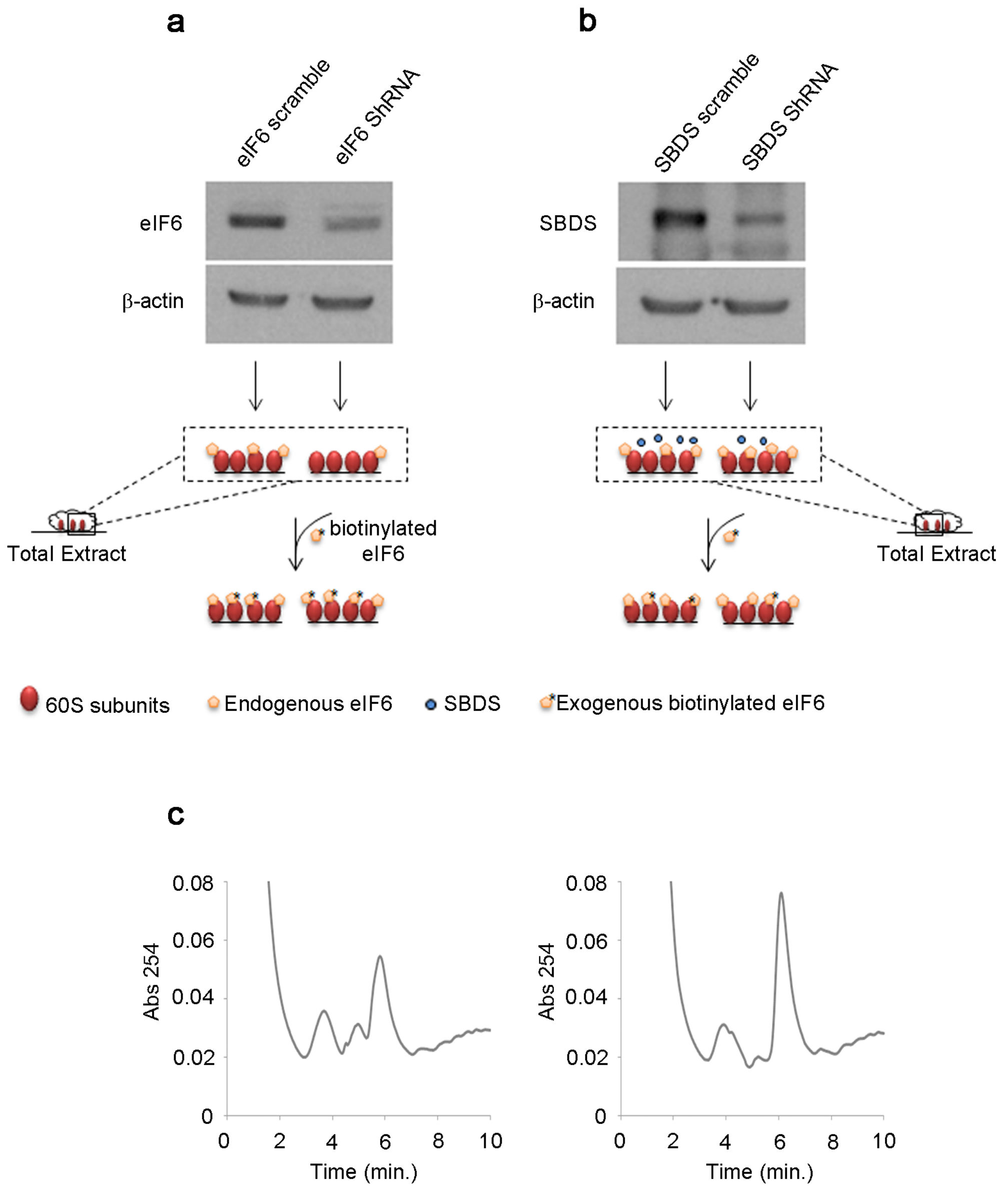

Supplementary Figure 5. Representative Western blots for eIF6 and SBDS post silencing. (a,b) HeLa cells were infected with lentiviral vectors encoding either a negative control shRNA sequence (scramble) or an shRNA specific for eIF6 and SBDS. Lysates were subjected to Western blots for individual proteins as indicated. Controls show β-actin levels. (c). Polysomal profiles of REN cells without (left) or with (right) eIF6 shRNA shows the increase of 80S in the absence of eIF6, due to the loss of the antiassociation activity. All experiments were performed at least five times.

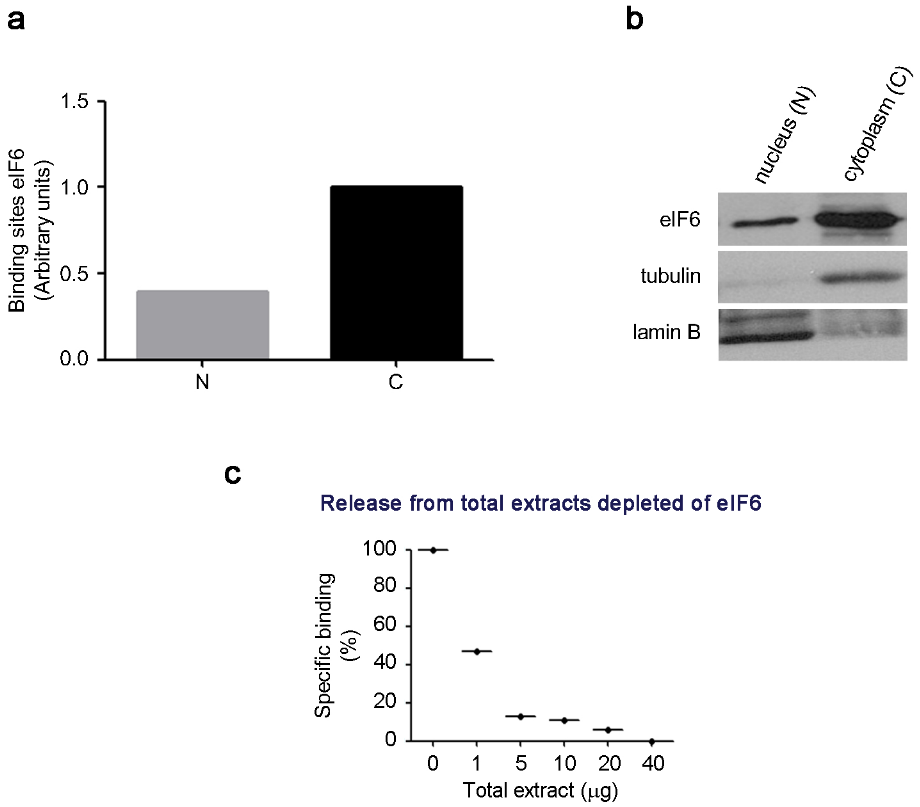

Supplementary Figure 6. Major number of binding sites for eIF6 are in the cytoplasm. (a) Nuclear (N) and cytoplasmic (C) fractionation, performed on Hek-293 cells, was used in iRIA to coat 96-well plate. Next, recombinant biotinylated eIF6 was added. Graphic represent the quantification of eIF6 total binding sites in the nucleus and in the cytoplasm. Data were normalized for number of cells (b) Western blots analysis of endogenous eIF6 fractionated into cytosolic and nuclear extracts. (c) Displacement curve of total extract deprived of eIF6 (blot, refer to Supplementary Figure 5) performed as in Figure 3f. All experiments were performed at least five times. Representative experiment is shown.
